# Dual-Specificity Phosphatases in Neuroblastoma Cell Growth and Differentiation

**DOI:** 10.3390/ijms20051170

**Published:** 2019-03-07

**Authors:** Caroline E. Nunes-Xavier, Laura Zaldumbide, Olaia Aurtenetxe, Ricardo López-Almaraz, José I. López, Rafael Pulido

**Affiliations:** 1 Biomarkers in Cancer Unit, Biocruces-Bizkaia Health Research Institute, Barakaldo, Bizkaia 48903, Spain; olaia.aurtenetxesaez@osakidetza.eus (O.A.); joseignacio.lopezfernandezdevillaverde@osakidetza.eus (J.I.L.); 2 Department of Tumor Biology, Institute for Cancer Research, Oslo University Hospital HF Radiumhospitalet, Oslo 0424, Norway; 3 Department of Pathology, Cruces University Hospital, University of the Basque Country (UPV/EHU), Barakaldo, Bizkaia 48903, Spain; laura.zaldumbideduenas@osakidetza.eus; 4 Pediatric Oncology and Hematology, Cruces University Hospital, Barakaldo, Bizkaia 48903, Spain; ricardo.lopezalmaraz@osakidetza.eus; 5 IKERBASQUE, Basque Foundation for Science, Bilbao 48011, Spain

**Keywords:** neuroblastoma, neuronal differentiation, dual-specificity phosphatases, MAP kinases, MAP kinase phosphatases, atypical dual-specificity phosphatases

## Abstract

Dual-specificity phosphatases (DUSPs) are important regulators of neuronal cell growth and differentiation by targeting proteins essential to neuronal survival in signaling pathways, among which the MAP kinases (MAPKs) stand out. DUSPs include the MAPK phosphatases (MKPs), a family of enzymes that directly dephosphorylate MAPKs, as well as the small-size atypical DUSPs, a group of low molecular-weight enzymes which display more heterogeneous substrate specificity. Neuroblastoma (NB) is a malignancy intimately associated with the course of neuronal and neuroendocrine cell differentiation, and constitutes the source of more common extracranial solid pediatric tumors. Here, we review the current knowledge on the involvement of MKPs and small-size atypical DUSPs in NB cell growth and differentiation, and discuss the potential of DUSPs as predictive biomarkers and therapeutic targets in human NB.

## 1. Introduction

Neuroblastoma (NB) is the most common malignancy diagnosed in the first year of life, with an average age at diagnosis of 18 months, and constitutes the most frequent extracranial solid tumor in infants, accounting for about 7% of total pediatric tumors. Although novel treatments have improved the survival of NB patients (about 80% 5-year survival), the high-risk forms of NB are the major cause of pediatric cancer death, rendering about 15% of pediatric cancer mortality [1,2]. NB is a neuroendocrine embryonal malignancy that derives from developing sympathetic neuronal cells from the peripheral nervous system, primarily from the adrenal gland medulla (about 40%), but also from the paraspinal sympathetic ganglia from the thorax, abdomen, neck, and pelvis [3,4,5]. NB arising from adrenal medulla display the worst prognosis, whereas those from the thorax, neck, and pelvis display better prognoses [6]. The current major criteria for staging and classification of NB cases are age, tumor histology subtype and differentiation, tumor spreading (about 20% of NB tumors are disseminated at diagnosis, with bone, bone marrow and liver as the more frequent metastatic niches), ploidy status and segmental chromosome aberrations, and *MYCN* (encoding an oncogenic transcription factor; about 25% of cases) and *ALK* (encoding an oncogenic receptor tyrosine kinase (RTK); about 3% of cases) genes amplification (*MYCN* and *ALK* are physically linked at 2p24-2p23). Children with *MYCN* amplification regardless of age, those older than 12 months with disseminated tumors, or those older than 18 months with unfavorable histology, are considered to be part of the high-risk NB group (about 40% of cases). Very low-, low-, and intermediate-risk NB patients show a 5-year survival of 90–95%, whereas high-risk NB patients show a 5-year survival of 40–50% [7,8,9]. The risk group determines the therapeutic treatment of NB patients, from observation or surgery alone for low-risk patients to multimodal therapy for high-risk patients. Multimodal therapy includes surgery, chemotherapy and radiotherapy, myeloablative therapy followed by bone marrow autologous transplantation, and immunotherapy and retinoic acid (RA)-based maintenance therapies. Current high-risk NB clinical trials are testing the efficacy of drugs targeting specific drivers of NB or major pro-oncogenic proteins. These include, among others, ALK and other RTKs, components of MYCN downstream pathways such as ornithine decarboxylase (ODC1), and components of the PI3K/AKT/mTOR and RAS-ERK1/2 MAPK signaling pathways [10,11,12,13,14].

Familial NB accounts for 1–2% of NB cases, with two major genes showing germline mutations in association with the disease: the *ALK* RTK gene, which is mainly expressed in the developing nervous system [15,16]; and the *PHOX2B*, encoding a transcription factor essential in neuronal differentiation and development of the autonomic nervous system, which is mutated in patients with congenital central hypoventilation syndrome (CCHS) [17]. Comprehensive analyses of the somatic mutational status of human cancers have revealed that the mutational burden of NB tumors is relatively low, compared with other cancers, a property which is shared by most pediatric tumors [18,19,20,21]. In addition, evidence for the existence of sporadic NB susceptibility genes has been recently obtained by several genome-wide association (GWA) studies on NB tumors, although the applicability of these findings has yet to be translated to the clinics [22,23,24]. In a GWA study, single nucleotide polymorphisms within the *DUSP12* gene, which encodes a large-size atypical DUSP proposed to target MAPKs, were associated with low-risk NB [25,26]. In this review, we summarize the current knowledge on the role of MAPK phosphatases (MKPs) and related small-size atypical DUSPS in NB, and their potential as NB biomarker and drug targets.

## 2. Neuroblastoma Cell Growth and Differentiation

**Figure 1:**
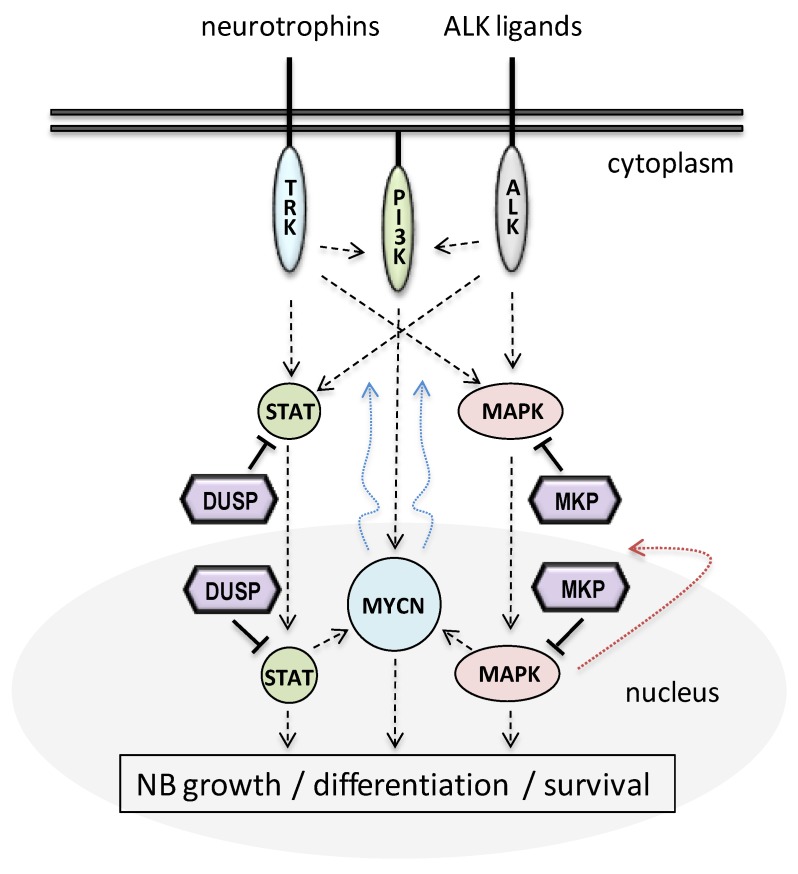
Schematic depiction of the major pathways involved in signaling through the ALK-MYCN axis in neuroblastoma (NB). ALK signals downstream mainly through the RAS-ERK1/2 MAP kinase (MAPK) pathway, as well as through the PI3K/AKT and JAK/STAT pathways, resulting in increased *MYCN* transcription and cell growth (straight dashed lines). Signaling through Trk neurotrophin receptors is also shown. MYCN transcriptional activity positively feeds the pathway by promoting *ALK* transcription (curved blue dotted lines), whereas transcriptional activity mediated by the MAPK nuclear effectors negatively feed-back the MAPK pathways by promoting the transcription of MAPK phosphatases (MKP) genes (curved red dotted line). Straight solid lines indicate direct dephosphorylation of protein substrates by MKPs or by other dual-specificity phosphatases (DUSPs). Dephosphorylation of MAPKs by MKPs is well documented, whereas evidence on the dephosphorylation of STATs or Trks by other DUSPs is limited. See text for more details.

NB can be considered a neural crest-related developmental tissue disease in which alterations in neuronal differentiation, driven by the unbalanced action of pro-proliferative and pro-differentiation factors on the maturation and migration of neural crest cells and neuroblasts, play a fundamental etiologic role. Highly differentiated NB tumors have a favorable clinical outcome, and spontaneous regression linked to neuroblast apoptosis is frequent even in metastatic cases [5,27,28,29]. *MYCN* amplification, the major hallmark of high-risk NB, associates with poorly differentiated NB tumors [30,31,32], and signaling through ALK favors proliferation and/or survival depending on the maturity of the neural cells [33,34]. In addition, activation of the tyrosine kinase neurotrophin receptors TrkA and TrkB leads to apoptotic/differentiation neuroblast responses or to survival/proliferative effects, respectively [35,36,37]. Several animal models suitable to the study of NB differentiation and transformation have been generated, mainly centered in *MYCN* amplification and ALK hyperactivation [38,39]. NB cell differentiation can also be triggered in vitro by culturing NB cells in the presence of differentiation factors, such as retinoids (retinoic acid, RA), phorbol esters (phorbol 12-myristate 13-acetate, PMA), and neurotrophins (nerve growth factor, NGF; brain-derived neurotrophic factor, BDNF) [40,41]. Human and rodent cell lines commonly used to study NB cell differentiation include SH-SY5Y and other derivatives from the SK-N-SH cell line, IMR-32, SMS-KCNR (all NB; human), Neuro2A (NB; mouse), PC12 (pheochromocytoma; rat), and P19 (embryonic teratocarcinoma; mouse) cell lines, among others. Extensive experimental work using these model systems has provided a picture in which the major signaling pathways involved in the molecular effects of MYCN and ALK in NB are the RAS/MAPK, PI3K/AKT, and JAK/STAT pathways [41,42,43,44] (Figure 1).

Genes related to the RAS-ERK1/2 MAPK pathway display somatic alterations in about 5% of sporadic primary NB tumors, with a much higher percentage of alterations (especially in *ALK*, *NRAS* and *NF1* genes) in relapsed NB samples obtained after chemotherapy [45,46,47]. This makes the RAS-ERK1/2 axis a potential responsive pathway for therapeutic intervention in NB. MEK1/2 or RAF-MEK1/2 pharmacological inhibitors displayed good inhibitory growth and survival effects, and decreased pERK1/2 levels, on NB cell lines with mutations in the RAS-ERK1/2 axis and high ERK1/2 activation under basal conditions [48,49,50,51]. Interestingly, combined use of a MEK1/2 inhibitor and RA resulted in increased differentiation and inhibitor sensitization of NB cells [52]. On the other hand, ALK-addicted NB cells responded to MEK1/2 inhibitors with an increase in survival and proliferation, which was associated with ERK5 activation through the PI3K/AKT pathway [53,54]. This highlights the importance of precise molecular segregation of NB patients before testing MEK1/2-inhibition in combination with other therapies, and illustrates the key role of distinct ERK kinases in mediating the efficacy of MEK1/2 inhibitors in NB. The influence of alterations in JNK and p38 MAPK pathways in NB cell growth has been investigated less. Higher sensitivity to apoptotic stimuli has mainly been reported in NB cell lines upon pharmacological inhibition of JNKs or p38s, although an anti-apoptotic outcome after inhibition of these MAPKs has also been reported [55,56,57,58,59] (Table 1). A complex scenario emerges in NB in which the specific contribution of the effectors from the distinct MAPK pathways may differentially drive NB cell growth, differentiation, and apoptosis, with variable consequences in the response to chemotherapies. As it has been proposed for other cell types, it is possible that a locally- and timely-regulated MAPK activation is critical for the response of NB cells to apoptotic or stress conditions. The interference of MAPK function by the inhibition of MAPK activators or inactivators (including phosphatase inactivators) is an open therapeutic possibility that deserves further exploration in NB.

Finally, the involvement of the pro-survival PI3K/AKT and JAK/STAT pathways in NB has also been disclosed in a variety of studies, sometimes acting in coordination with MAPK activities; the inhibition of the effectors of these pathways as a NB therapeutic option is under scrutiny [44,87,88,89,90]. A functional ALK-MYCN axis operates in NB that positively controls cell growth through coordinated integration of these major signaling pathways [15,91,92] (Figure 1).

## 3. DUSPs in NB Cell Growth and Differentiation

**Figure 2:**
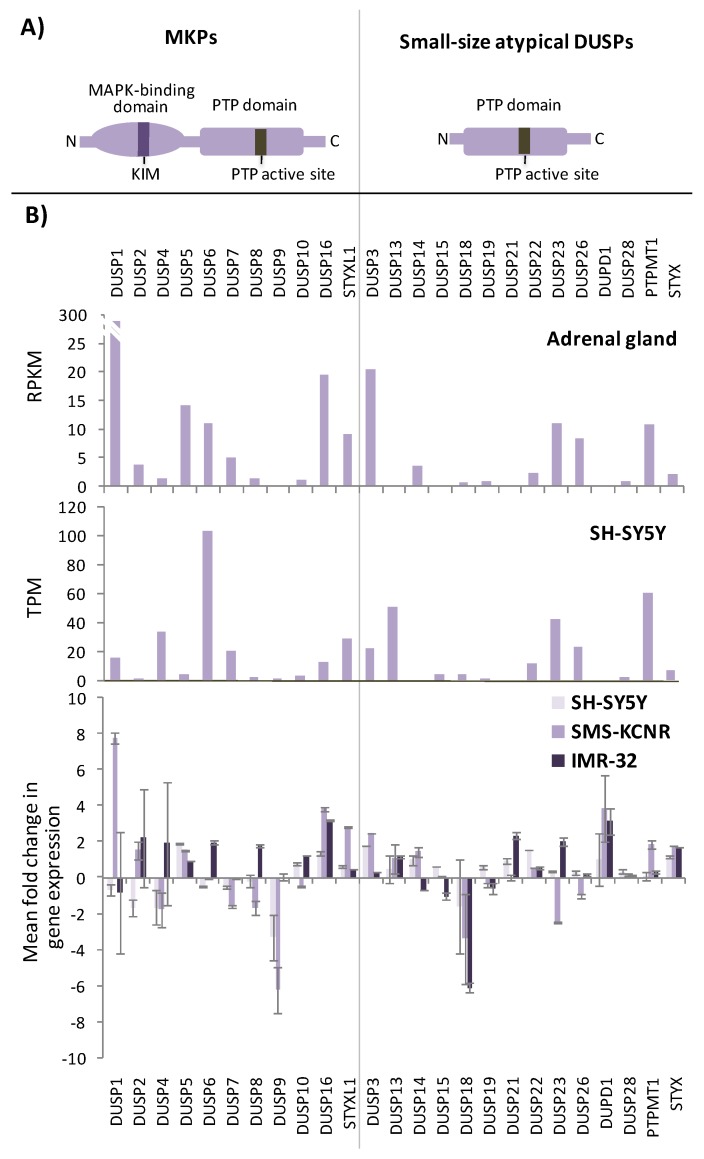
(**A**) Schematic depiction of the domain composition of MKPs and small-atypical DUSPs. KIM—kinase interaction motif; PTP—protein tyrosine phosphatase. (**B**) Upper panel: mRNA expression of MKPs and small-atypical DUSPs in adrenal gland. Data from GTEx (Genotype-Tissue Expression) data sets. RNA-seq data is reported as median RPKM (reads per kilobase per million reads mapped) (https://www.proteinatlas.org/). Middle panel: mRNA expression of MKPs and small-atypical DUSPs in SH-SY5Y human NB cells; data from the Human Protein Atlas. RNA-seq data is reported as median TPM (transcripts per kilobase of exon per million reads) (https://www.proteinatlas.org/). Bottom panel: mRNA expression analysis of MKPs and small-atypical DUSPs from SH-SY5Y, SMS-KCNR, and IMR-32 human NB cell lines treated with retinoic acid (RA). Cell lines were kept untreated or were treated for 10 days with RA, mRNA was extracted and RT-qPCR was performed using a set of MKP and small-atypical DUSP primers, as described in Reference [67]. Relative mRNA expression values are shown in Log_2_ as fold change +SD of treated cells *versus* untreated cells, from at least two independent experiments. Mean fold change above 2 or below −2 was considered significant. MKP mRNA expression data from bottom panel has been previously published [67].

DUSPs constitute a heterogeneous group of non-transmembrane enzymes within the class I Cys-based protein tyrosine phosphatase (PTP) family. They have in common the presence of a single catalytic PTP domain, which in the members of several DUSP subfamilies, has the dual capability to dephosphorylate both Ser/Thr- and Tyr-phosphorylated residues (pSer/pThr, pTyr) in proteins. Enzymes from other DUSP subgroups, however, have RNA, lipids, or other biomolecules as their major substrates [93,94,95,96]. Here, we will focus on the MAPK phosphatase PTP subfamily (MKPs; 11 genes in humans) and the MKP-related small-size atypical DUSPs (15 genes in humans) (Figure 2), two groups of DUSPs with high potential as anti-cancer drug targets and as regulators of NB cell growth and differentiation [97,98]. With the exception of the phosphatase inactive MKP STYXL1, MKPs are specialized in the selective dephosphorylation of the Thr and Tyr regulatory residues from the distinct MAPKs (ERKs, p38s, and JNKs), a group of Ser/Thr kinases that shuttle between the cytoplasm and the nucleus and have major physiologic roles as modulators of cell growth, differentiation, and apoptosis upon changes in extracellular cues. Accordingly, alterations in the outcome of the MAPK pathways have a notable impact on human disease [99,100,101,102,103]. MKPs are composed of a catalytic DUSP-PTP domain and a regulatory MAPK-binding domain, which is essential to control the specificity in the binding and dephosphorylation of the distinct MAPKs (Figure 2A). In addition to their role in MAPK dephosphorylation, which results in MAPK catalytic inactivation, MKPs also directly regulate the nuclear-cytoplasmic shuttling of MAPKs upon the binding of their MAPK-binding domains [104,105,106]. MKPs expression, subcellular localization, and function is highly regulated during physiologic and pathologic processes, and MKPs gene expression is induced in many cases upon activation of the specific MAPK pathways under their regulation. In consequence, expression of different MKPs has been associated with several forms of human cancer [107,108,109]. Small-size atypical DUSPs lack the regulatory MAPK-binding domain and constitute small enzymes, among which DUSP3 is the one more intensively studied [110] (Figure 2A). Most of small-size atypical DUSPs dephosphorylate pSer/pThr and pTyr from proteins (including MAPKs, STATs, and RTKs), whereas some of these enzymes have as physiologic substrates other biomolecules or, in the case of the small-size atypical DUSP STYX, are phosphatase inactive [111,112].

The functional role of MKPs and small-size atypical DUSPs as physiologic MAPK inactivators advocates for their high potential as important players in NB cell growth and differentiation (Figure 1). In Figure 2B (upper and middle panels), the mRNA expression profiles of MKPs and small-size atypical DUSPs in the adrenal gland (the more common source of NB cells) and SH-SY5Y NB cells (the more studied human NB cell line) are shown. In Figure 2B (bottom panel), the changes in the mRNA expression of MKPs and small-size atypical DUSPs from three human NB cell lines (SH-SY5Y, SMS-KCNR, and IMR-32) undergoing retinoic acid (RA)-induced differentiation are shown. Different RA-regulated expression patterns of these genes can be observed, suggesting a complex and cell-specific rearrangement of DUSPs gene expression during NB cell differentiation. Following is an account on the expression and function of MKPs and small-size atypical DUSPs in NB, and a summary of the information is provided in Table 1. MKPs have been grouped according to amino acid sequence conservation, subcellular localization and substrate specificity, as reported in References [98,113].

### 3.1. MKPs in NB Cell Growth and Differentiation

#### 3.1.1. DUSP1, DUSP4, and DUSP5

DUSP1 (MKP1), DUSP4 (MKP2), and DUSP5 constitute a group of highly transcriptionally inducible MKPs with nuclear localization and distinct MAPK substrate specificity. DUSP1 mainly dephosphorylates JNKs and p38s, whereas DUSP4 and DUSP5 are more specific for ERK1/2 as a substrate [114,115,116]. DUSP1 is the founder of the MKP family and displays ubiquitous tissue distribution. DUSP1 has been widely involved in human disease, including immunological, inflammatory and cardiovascular diseases, cancer, and developmental nervous system diseases [117,118,119,120,121,122]. In human NB cell lines, DUSP1 has been shown to facilitate apoptotic processes, suggesting a negative role for this MKP in NB cell survival. H_2_O_2_ treatment of SH-SY5Y cells triggered apoptosis which was accompanied by early reduction and late induction of DUSP1 protein content, in inverse correlation with pERK1/2 levels, and siRNA suppression of DUSP1 expression attenuated H_2_O_2_-induced cell death [60]. It would be important to determine the contribution of DUSP1 mRNA transcription to increasing DUSP1 levels in SH-SY5Y cells during long-term H_2_O_2_ treatment. In this regard, treatment of SH-SY5Y cells with silver nanoparticles (AgNP) inducing neuronal differentiation increased reactive oxygen species (ROS) generation, which was accompanied by decreased mRNA levels in several DUSPs, including DUSP1, and increased pAKT and pERK1/2 levels [61,123]. In addition, carbachol treatment of SH-SY5Y cells resulted in rapid ERK1/2 activation and increased nuclear DUSP1 protein content [62], whereas long-term stimulation with the differentiating agent RA caused up-regulation of DUSP1 mRNA in SMS-KCNR NB cells, but not in SH-SY5Y cells [67]. Interestingly, CD133+ stem-cell subpopulations from the human NB cell lines SK-N-SH and SK-N-BE displayed high resistance to pro-apoptotic chemotherapeutic agents, in association with DUSP1 protein low expression levels and high phosphoERK1/2 (pERK1/2) and phosphop38 (pp38) content, suggesting that DUSP1 expression, at least in some NB cell populations, may facilitate chemotherapy efficacy [63]. Treatment of GH4C1 rat neuroendocrine cells with epidermal growth factor (EGF) or thyrotropin-releasing hormone (TRH) triggered DUSP1 mRNA translation [65], and P19 mouse embryonic stem cells (ESCs), induced to neuronal differentiation by RA treatment, also displayed increased nuclear DUSP1 protein content, coinciding with ERK1/2 inactivation [64]. On the other hand, an anti-apoptotic role has also been proposed for DUSP1 in NB cell lines in association with its action on JNKs. Hypoxia/re-oxygenation of N1E-115 mouse NB cells resulted in up-regulation of mRNA and protein DUSP1 levels, although JNK activation was not suppressed under these conditions. However, the siRNA knock-down of DUSP1 in N1E-115 cells induced to differentiate in the absence of serum resulted in increased JNK activation and apoptotic cell death, whereas DUSP1 over-expression caused the opposite effects [68]. These findings support the notion that the inhibitory action of nuclear DUSP1 on specific MAPKs at specific time-points could be a determinant of the survival of NB cells challenged with pro-apoptotic or pro-differentiation chemotherapies.

DUSP4 has been regarded as a potential tumor suppressor, as well as a promoter of chemotherapy resistance, in several human cancers [124,125,126,127,128]. DUSP4 expression augmented during RA-dependent neuronal differentiation of mouse ESCs, and shRNA silencing of DUSP4 expression in mouse ESCs decreased neuronal differentiation, in association with elevated pERK1/2 content and alterations in calcium homeostasis [69]. In contrast, DUSP4 mRNA was downregulated in SH-SY5Y cells subjected to neuronal differentiation by AgNP or RA [61,67]. In addition, human SK-N-AS NB cells subjected to pharmacologic ALK inhibition displayed mRNA downregulation of several ERK1/2 inactivators, including DUSP4, DUSP5, and DUSP6, whereas overexpression of hyperactive ALK caused upregulation of these MKPs [70]. This is suggestive of a negative ERK1/2 feed-back regulation, which could be operative during NB progression. Whether regulation of DUSP4 expression during NB cell growth is related to malignancy deserves further studies.

**Figure 3:**
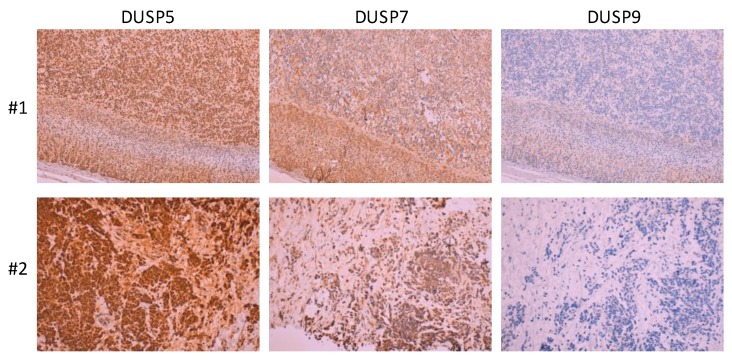
Immunostaining of selected MKPs from tissue sections from two NB tumors (#1: Stage I, unknown *MYCN* status, low-risk; #2: Stage IV, metastatic, *MYCN* amplification, high-risk). Representative staining patterns are shown. DUSP5 and DUSP7 mostly showed high-moderate immunoreactivity, whereas DUSP9 mostly displayed low immunoreactivity (magnification is ×100). Immunostaining was performed as described in [67].

DUSP5, together with DUSP6, is an ERK1/2-specific MKP whose transcription is induced in an ERK1/2-dependent manner [129,130,131,132]. Some reports attribute tumor suppressor activities to DUSP5, and a dual role for this MKP in carcinogenesis, depending on the tissue and cellular context, has been proposed [109]. DUSP5 mRNA was upregulated on several human NB cell lines subjected to RA-induced differentiation. In SH-SY5Y cells, DUSP5 mRNA upregulation correlated with ERK1/2 activation conditions, illustrating that upregulation of ERK1/2-specific MKPs in NB cells is concomitant to the activation of ERK1/2 pathway, and DUSP5 siRNA knock-down resulted in increased cell proliferation. Importantly, high expression of DUSP5 protein was relatively frequent and also correlated with pERK1/2 expression in NB tumors, in association with poor patient prognosis, likely as a surrogate marker of ERK1/2 activation [67] (Figure 3). In another study, the expression in human cancer cells of DUSP5 and the DUSP5 pseudogene DUSP5P1 was compared, showing high ratios of DUSP5P1/DUSP5 expression in cancer cell lines, including NB cell lines, when compared to normal tissues [71]. In addition, DUSP5P1 expression has been found to be associated with poor prognosis in acute myeloid leukemia [133]. Further investigation is required to determine the role of DUSP5P1 in regulating DUSP5 function, and whether DUSP5P1 expression correlates with the prognosis of or tumor remission in NB.

#### 3.1.2. DUSP6, DUSP7, and DUSP9

DUSP6 (MKP3), DUSP7 (MKPX), and DUSP9 (MKP4) are cytoplasmic MKPs with specificity towards ERK1/2 dephosphorylation. Similar to DUSP5, DUSP6 is inducible by growth and differentiating agents that activate the ERK1/2 pathway [132,134,135], and DUSP6 is also reported to play a tissue-specific dual tumor suppressive or pro-oncogenic role [105,136]. DUSP6 mRNA is induced with NGF- or fibroblast growth factor (FGF)-mediated neuronal differentiation in PC12 rat pheochromocytoma cells [72,73,74], as well as with RA-mediated neuronal differentiation of SH-SY5Y, BE(2)-C, and IMR-32 human NB cells [67,75]. On the other hand, SH-SY5Y cells challenged with H_2_O_2_ displayed reduced DUSP6 protein levels, which was associated with a higher pERK1/2 content and cell death [76]. This effect is likely to be mediated by caspase-3 cleavage of DUSP6, as described for other human cancer cell lines [104]. Besides, co-silencing of DUSP6 and RGS16 (a negative regulator of Ras G proteins also induced by RA) in SH-SY5Y cells increased pERK1/2 levels and decreased the proliferation arrest caused by RA in these cells [75]. Differentiation of P19 mouse cells with RA also increased DUSP6 protein levels dependent on ERK1/2 activation. Interestingly, DUSP1 protein accumulation in these conditions was independent on ERK1/2 activation [64]. As mentioned, DUSP6 mRNA induction can be triggered in NB cells by signaling through hyperactive ALK [70], and *DUSP6* transcription can be repressed by MYCN in BE(2)-C and LAN-1 NB cells [77]. Together, these findings illustrate the complexity in the negative and positive ERK1/2 regulatory feed-back loops that operate in NB cells by regulation of *DUSP6* gene transcription and DUSP6 protein stability. They prompt further investigations of the potential of DUSP6 as a modulator of NB malignancy.

Expression of both DUSP7 and DUSP9 protein has been detected in NB tumor samples, although, in the case of DUSP9, with a low frequency [67] (Figure 3). Human SH-SY5Y and SMS-KCNR cells, as well as mouse J1 ESCs, displayed downregulation of DUSP9 mRNA upon RA-induced differentiation, which in the case of mouse J1 cells was confirmed at the protein level [67,78].

#### 3.1.3. DUSP8, DUSP10, and DUSP16

DUSP8, DUSP10 (MKP5), and DUSP16 (MKP7) are MKPs with substrate specificity towards JNKs and p38s MAPKs. These three MKPs are larger than the rest of the MKPs, with N- or C-terminal extensions in their amino acid sequences. DUSP10 and DUSP16 have been involved in autoimmunity and inflammation processes, and little is known about the physiological function of DUSP8. Interestingly, *DUSP16* gene loss caused perinatal lethality in mice, associated with hydrocephalus, brain overgrowth by expansion of neural progenitors, and increased midbrain pp38 content [79]. RA treatment has been reported to upregulate DUSP8 and DUSP10 in mouse J1 ESCs, and DUSP16 mRNA in human NB cell lines [67,78].

### 3.2. Small-Size Atypical DUSPs in NB Cell Growth and Differentiation

DUSP3 (VHR) is the prototype of small-size atypical DUSPs, and displays substrate specificity towards ERK1/2 and JNKs. However, some other proteins, including STAT5, FAK, growth factor receptors, and nuclear proteins regulating DNA damage repair, have been shown to be tyrosine-dephosphorylated by DUSP3 and have been proposed as DUSP3 physiological substrates [110,137]. As mentioned for other MKPs, DUSP3 mRNA was upregulated in SH-SY5Y cells differentiated in the presence of AgNP [61], as well as on SH-SY5Y or SMS-KCNR cells differentiated by RA (Figure 2B, bottom panel), but the potential role of DUSP3 in NB cell growth and differentiation remains unexplored.

DUSP13A and DUSP13B are two small-size atypical DUSPs encoded in the same gene and generated by the use of alternative open reading frames, whose physiologic substrate specificity is poorly known [138]. DUSP13A (MDSP) was found to be a potential regulator of apoptosis in human SK-N-SH NB cells in a phosphatase-independent manner, by virtue of its physical association with the p38- and JNK-activator protein apoptosis signal-regulated kinase 1 (ASK1). The knock-down of DUSP13A decreased the phosphorylation and activation of ASK1 [80].

DUSP23 (VHZ) has been reported to affect MAPKs activation both by phosphatase-dependent and -independent mechanisms [139,140], as well as the Ser- or Tyr-phosphorylation status of other proteins, including β-catenin and the transcription factor GCM1 [141,142]. DUSP23 mRNA and proteins were upregulated in RA-differentiated mouse ESCs, and siRNA knock-down of DUSP23 in these cells decreased neuronal differentiation, together with increased pp38 and decreased pERK1/2 and phosphoJNK (pJNK), likely by a combination of catalytic and scaffolding properties [78]. Noticeably, analysis of the methylation status of the *DUSP23* gene in NB tumors revealed higher methylation in *MYCN*-amplified tumors. In addition, DUSP23 mRNA was at lower levels in NB patients with poor outcomes, when compared to patients free of disease [81]. This suggests a tumor suppressive role for DUSP23 in NB. It would be important to analyze DUSP23 protein expression in these groups of NB patients.

DUSP26 (MKP8) was originally identified as a DUSP mainly expressed in embryonal cancers and displaying substrate specificity towards p38 in cells [143]. Subsequently, DUSP26 was also found in neuroendocrine tissues, and induced by NGF-stimulation in PC12 cells [82], as well as by RA in mouse ESCs [78]. DUSP26 overexpression in PC12 cells decreased EGFR and pAKT levels, which resulted in the suppression of NGF- and EGF-induced signaling. This effect was dependent on DUSP26 phosphatase activity, but was not associated with changes in MAPKs activation. Opposite consequences were observed with DUSP26 knock-down [82,83]. Additional studies in PC12 cells and in the zebrafish model sustain the possibility that DUSP26 could target for dephosphorylation specific receptor tyrosine kinases, including TrkA and FGFR1 [144]. In IMR-32 cells, DUSP26 has been proposed to increase N-cadherin-mediated cell-cell adhesion by dephosphorylation of the KIF3 motor complex component Kap3 [145]. A suppressive role of cell proliferation by DUSP26 targeting non-MAPK substrates has also been unveiled in epithelial cancer cells [84]. In contrast, an oncogenic role has been proposed for DUSP26 in human NB cells on the basis of DUSP26-mediated inhibition of p53 activity by specific Ser dephosphorylation and resistance to doxorubicin-induced apoptosis. Importantly, high-risk NB tumors displayed the highest levels of DUSP26 protein expression from a limited number of samples [85]. DUSP26 knock-down in SH-SY5Y cells inhibited cell growth in vitro and in xenograft mice, and this could be summarized by the pharmacological inhibition of DUSP26, which also increased phosphop53 (pp53) and pp38 levels [86]. Together, these results argue for both MAPK-dependent and -independent effects of DUSP26 in NB cell growth. It would be necessary to further define the oncogenic or tumor suppressive functions of DUSP26 in human NB before pointing to DUSP26 as a target for inhibition in these types of cancers.

## 4. Concluding Remarks

DUSPs have been proposed as cancer biomarkers in several human cancers, and recent advances in the understanding of the biology of DUSPs have made these enzymes potentially targetable molecules in cancer therapy: (1) DUSPs regulate cell growth, differentiation, and apoptosis through dephosphorylation of key effector proteins from major intracellular signaling pathways; (2) DUSPs present a well-defined catalytic mechanism towards well-defined substrates, especially those in the MAPK family; and (3) DUSPs activities can be efficiently repressed by small molecule inhibitors [96,98,108,109,146]. Signaling through the ALK-RAS-ERK1/2 MAPK pathway associates with NB tumor progression and relapse. Since the expression of some ERK1/2-specific MKPs, such as DUSP5 or DUSP6, is upregulated upon ERK1/2 activation, their expression in NB could be used as a surrogate marker of ALK and ERK1/2 activation status. In such scenario, inhibition of ERK1/2-MKPs is not desirable. Instead, increased activity of ERK1/2-MKPs would be therapeutically advantageous under the conditions of RAS-ERK1/2 pathway hyperactivation in NB, especially in high-risk NB committed to undifferentiation. On the other hand, the variable effects of JNK and p38 MAPKs on NB cell apoptosis endorse the possibility of a beneficial use of inhibitors of JNK- or p38-specific MKPs, such as DUSP1, DUSP8, DUSP10, or DUSP16, but only in NB cases in which the activity of JNKs or p38s favors tumor chemosensitivity. Finally, the possibility that specific small-atypical DUSPs directly target upstream components in the MAPK pathways, or receptor or effector proteins in other NB cell growth/survival pathways, such as Trks or STATs, deserves further exploration. A precise molecular definition of the pathways involved in NB cell growth dependence and drug resistance in the distinct groups of NB patients is desirable. This would help to bring into practice NB therapies based on MKPs or small-atypical DUSPs functional interference.

## Figures and Tables

**Table 1:** MKPs and small-size atypical DUSPs in neuroblastoma cell growth and differentiation.

Gene/Protein	MAPK Substrates ^1^ Localization	Alterations in NB Cell Lines and NB Tumors
DUSP1/MKP1	JNK, p38 > ERK Nuclear	-SH-SY5Y cells: *DUSP1 KD:* ↓ H_2_O_2_-induced apoptosis, ↑ pERK1/2 [60]; ↓ DUSP1 mRNA upon AgNP treatment [61]; ↑ DUSP1 upon carbachol stimulation [62]. -SK-N-SH and SK-N-BE cells: ↓ DUSP1 protein in CD133+ cells resistant to chemotherapy [63]. -P19 cells: ↑ DUSP1 protein upon RA differentiation [64]. -GH4C1 neuroendocrine cells: ↑ DUSP1 upon TRH and EGF stimulation [65,66]. -SMS-KCNR cells: ↑ DUSP1 mRNA upon RA-induced differentiation [67]. -N1E-115 cells: ↑ DUSP1 upon hypoxia/re-oxygenation; *DUSP1 KD:* ↑ pJNK, ↑neuronal death; *DUSP1 OE:* ↓ pJNK, ↓ neuronal death [68].
DUSP4/MKP2	ERK, JNK > p38 Nuclear	-SH-SY5Y cells: ↓ DUSP4 mRNA upon AgNP treatment [61]. -Mouse ESCs: *DUSP4 KD:* ↓ neuronal differentiation, ↑ pERK1/2; ↑ DUSP4 in RA-induced differentiation [69]. -SH-SY5Y, KCNR cells: ↓ DUSP4 upon RA-induced differentiation [67]. -SK-N-AS cells: ↓ DUSP4 mRNA upon ALK inhibition, ↑ DUSP4 mRNA upon mutant ALK OE [70].
DUSP5	ERK Nuclear	-SiMa, Kelly, SH-SY5Y, CHP-134 cells: ↑ DUSP5P1/DUSP5 ratio compared to normal cells [71]. -SH-SY5Y, SMS-KCNR, IMR-32 cells: ↑ DUSP5 mRNA upon RA-induced neuronal differentiation; SH-SY5Y cells: ↑ DUSP5 mRNA upon EGF and PMA stimulation; *DUSP5 KD:*↑ proliferation [67]. -SK-N-AS cells: ↓ DUSP5 mRNA upon ALK inhibition, ↑ DUSP5 mRNA upon mutant ALK OE [70]. -NB tumors: DUSP5 protein expression associated with poor prognosis and pERK1/2 expression [67].
DUSP6/MKP3	ERK Cytoplasmic	-PC12 cells: ↑ DUSP6 mRNA in FGF and NGF-mediated neuronal differentiation [72,73,74]. -P19 cells: ↑ DUSP6 protein upon RA differentiation, dependent on ERK1/2 activation [64]. -SH-SY5Y, BE(2)-C cells: ↑ DUSP6 mRNA upon RA-induced differentiation; *DUSP6 KD* + *RGS16 KD* in SH-SY5Y cells: ↓ RA-mediated proliferation arrest [75]. -IMR-32 cells: ↑DUSP6 mRNA upon RA-induced differentiation [67]. -SH-SY5Y cells: ↓ DUSP6 protein upon H_2_O_2_ stimulation [76]. -SK-N-AS cells: ↓ DUSP6 mRNA upon ALK inhibition, ↑ DUSP6 mRNA upon mutant ALK OE [70]. -BE(2)-C, LAN-1 cells: ↓ DUSP6 by a MYCN-SIRT1 complex [77].
DUSP7/MKPX	ERK Cytoplasmic	-NB tumors: Positive immunostaining, no clinical associations [67].
DUSP8	JNK, p38 Cytoplasmic/nuclear	-Mouse J1 ESCs: ↑ DUSP8 in RA-induced neuronal differentiation [78].
DUSP9/MKP4	ERK > p38 Cytoplasmic	-Mouse J1 ESCs:↓ DUSP9 in RA-induced neuronal differentiation [78]. -SH-SY5Y, SMS-KCNR cells: ↓ DUSP9 mRNA upon RA-induced differentiation [67]. -NB tumors: Mostly negative immunostaining, no clinical associations [67].
DUSP10/MKP5	JNK, p38 Cytoplasmic/nuclear	-Mouse J1 ESCs: ↑ DUSP10 in RA-induced neuronal differentiation [78].
DUSP16/MKP7	JNK, p38 Cytoplasmic/nuclear	*-DUSP16 -/- mice*: hydrocephalus and brain overgrowth [79]. -SMS-KCNR, IMR-32 cells: ↑ DUSP16 mRNA upon RA-induced differentiation [67].
DUSP3/VHR	(ERK, JNK) Cytoplasmic/nuclear	-SH-SY5Y cells: ↓ DUSP3 mRNA upon AgNP treatment [61].
DUSP13A/MDSP	Cytoplasmic	-SK-N-SH cells: physical association with pro-apoptotic ASK1, independent of phosphatase activity; *DUSP13A KD:* ↓ ASK1 kinase activity [80].
DUSP23/VHZ	(ERK, JNK) Cytoplasmic/nuclear	-Mouse J1 ESCs: ↑ DUSP23 in RA-induced neuronal differentiation; *DUSP23 KD:*↓ neuronal differentiation, ↑ pp38, ↓ pERK1/2, ↓ pJNK [78]. -NB tumors: ↑ Methylation in *MYCN*-amplified tumors, ↓ DUSP23 mRNA in patients with poor outcome [81].
DUSP26/MKP8	(p38) Nuclear	-Mouse J1 ESCs: ↑ DUSP26 in RA-induced neuronal differentiation [78]. -PC12 cells: ↑ DUSP26 mRNA in NGF-induced differentiation; *DUSP26 KD:* ↑ EGFR, ↑ NGF-induced differentiation; *DUSP26 OE:* ↓ EGF-induced cell growth, ↓ NGF-induced differentiation, ↑ cisplatin-induced apoptosis, ↓ pAKT, ↓ EGFR [82,83]. -Human NB cell lines: ↓ DUSP26 mRNA compared to normal adrenal gland [84]. -IMR-32 cells: *DUSP26 KD:* ↑ doxorubicin-induced apoptosis, ↓ proliferation, ↑ pp53, ↑ pp38; SH-SY5Y cells: *DUSP26 KD:* ↓proliferation; SK-N-SH cells: *DUSP26 OE:* ↓ doxorubicin-induced apoptosis, ↓ pp53 [85,86]. -NB tumors: ↑ DUSP26 protein in high-risk NB tumors [85].

^1^ Note that substrate specificity towards MAPKs of small-size atypical DUSPs is debatable in some cases, and some effects on MAPK phosphorylation status have been reported to be likely mediated by scaffolding functions or by dephosphorylation of non-MAPK proteins. Abbreviations: AgNP—silver nanoparticles; ALK—anaplastic lymphoma kinase; EGF—epidermal growth factor; ESCs—embryonic stem cells; FGF—fibroblast growth factor; KD—knock-down; NB—neuroblastoma; NGF—nerve growth factor; OE—overexpression; PMA—phorbol 12-myristate 13-acetate; RA—retinoic acid; TRH—thyrotopin-releasing hormone; ↑—increase; ↓—decrease.

## Abbreviations

ALK	Anaplastic lymphoma kinase
BDNGF	Brain-derived neurotrophic factor
CCHS	Congenital central hypoventilation syndrome
DUSP	Dual-specificity phosphatase
EGFR	Epidermal growth factor receptor
ESC	Embryonic stem cell
FGFR	Fibroblast growth factor receptor
GWA	Genome-wide association
IFN	Interferon
KIM	Kinase interaction motif
MAPK	Mitogen-activated protein kinase
MKP	MAPK phosphatase
NB	Neuroblastoma
NGF	Nerve growth factor
PMA	Phorbol 12-myristate 13-acetate
PTP	Protein tyrosine phosphatase
RA	Retinoic acid
RTK	Receptor tyrosine kinase
TRH	Thyrotropin-releasing hormone
